# Supplementary figures and images for: DArTSNP based genetic diversity analyses in cassava (Manihote esculenta [Cranz]) genotypes sourced from different regions revealed high level of diversity within population
Source: PLoS One. 2025 Mar 19;20(3):e0308027. doi: 10.1371/journal.pone.0308027 (PMC11922271; doi:10.1371/journal.pone.0308027)

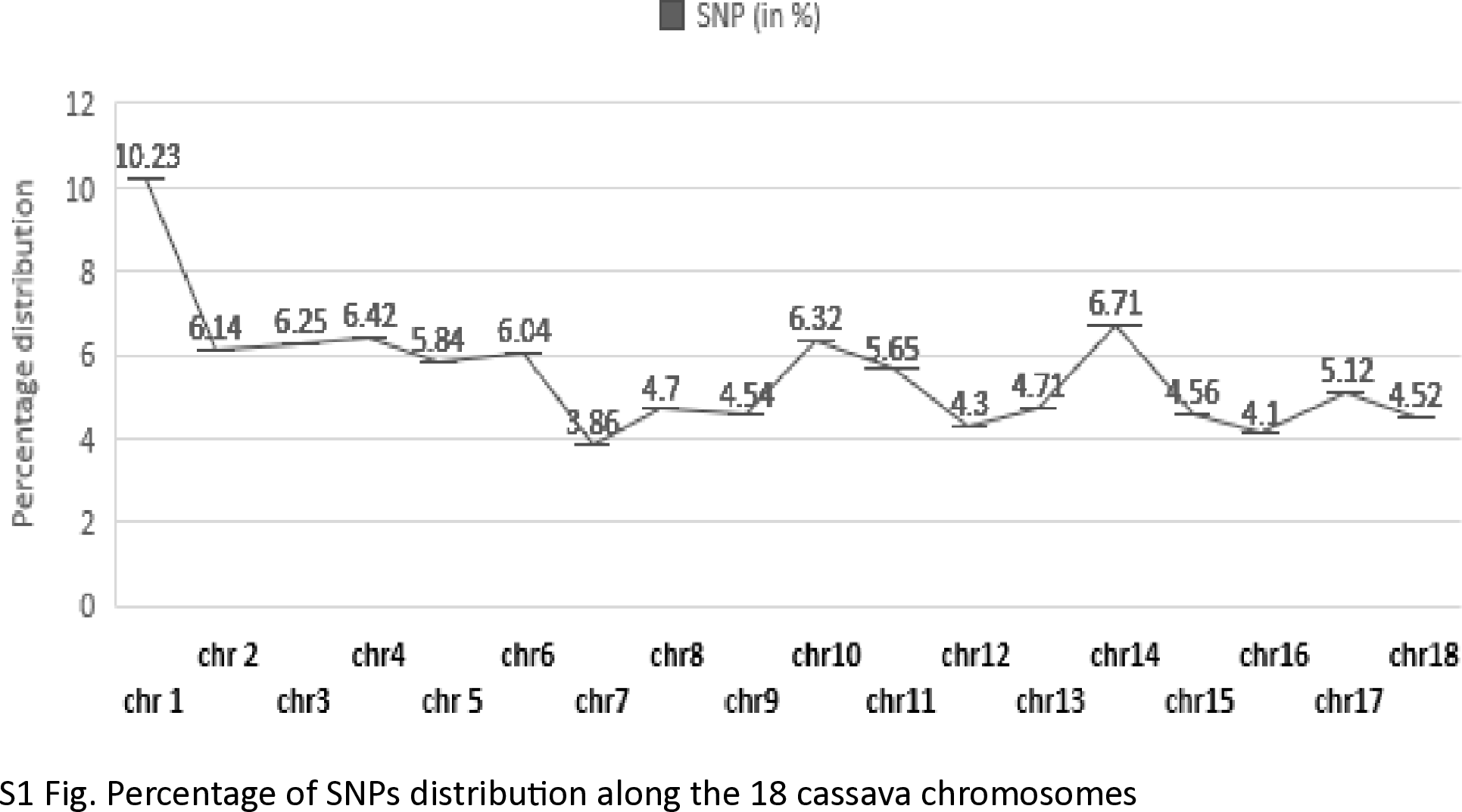

Supplement: S1 Fig — (TIF) [file pone.0308027.s001.tif]

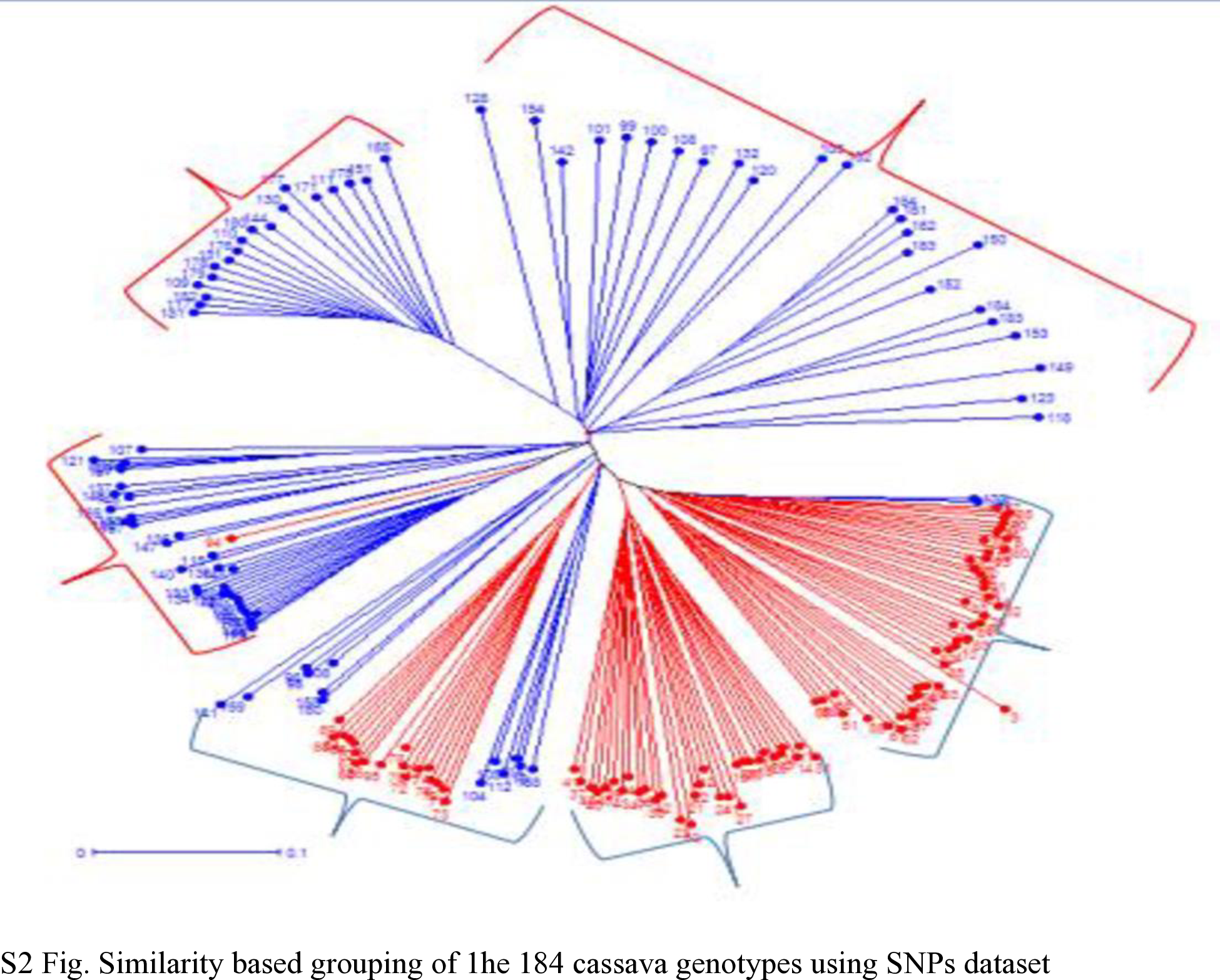

Supplement: S2 Fig — (TIF) [file pone.0308027.s002.tif]
