# Supplementary material for: DArTSNP based genetic diversity analyses in cassava (Manihote esculenta [Cranz]) genotypes sourced from different regions revealed high level of diversity within population
Source: PLoS One. 2025 Mar 19;20(3):e0308027. doi: 10.1371/journal.pone.0308027 (PMC11922271; doi:10.1371/journal.pone.0308027)
Supplement: Attachment — Submitted filename: MS ID PONE-D-24-29508.docx [file pone.0308027.s005.docx]

Dear editor,

I thoroughly examine the Manuscript ID: PONE-D-24-29508, entitled “DArTSNPs based genetic diversity analyses in cassava (*Manihote esculenta* [Cranz]) genotypes sourced from different regions revealed high level of diversity within population” which was submitted for Plos One journal for publication.

The manuscript was a well written article in all respects. The abstract, introduction, methodological aspects, presentation of results and discussion are sound and it is written in good language. Excellent work has been done to identify collection of casava accessions using molecular analysis in the country. I feel the subject is worthy enough for publication. Authors have clearly provided justification of genetic diversity and used appropriate statistical tools for this study for analysis. The conclusion is also sounds great. I did not find any major corrections to be made except some typographical errors as indicated below.

Abstract:

- AMOVA revealed higher variation within (91.3%) and between (8.7%) the study populations.

Text

- In text the reference should start with [1], not with the [13]. It needs to be in chronological order….please double check the references in text and reference list.
