# Supplementary material for: DArTSNP based genetic diversity analyses in cassava (Manihote esculenta [Cranz]) genotypes sourced from different regions revealed high level of diversity within population
Source: PLoS One. 2025 Mar 19;20(3):e0308027. doi: 10.1371/journal.pone.0308027 (PMC11922271; doi:10.1371/journal.pone.0308027)
Supplement: Attachment — Submitted filename: Response to reviewer.docx [file pone.0308027.s007.docx]

**Responses to reviewers’ comments**

First of all, I want to appreciate you for your time devotion and providing to me constructive comments and suggestion. I positively accepted your comments and my responses are as follow.

1. I rigorously revised for grammatical improvement, first introducing of abbreviation, and correcting appropriate formatting.
2. Concerning figure numbering and ordering as well, I have corrected figure citation in the body. I highlighted revised version in yellow color.
3. The age of the seedlings which was 15 days seedling after planting from which sampling carried out has been described. Also, where sequencing was carried out was well described and highlighted with yellow color and track change
4. Under result part, by accepting suggestion, Table 1 has been deleted. However, I think it does not matter if available because table summarize and we describe what we summarized.
5. For the software used, which its version was not mentioned has been revised and corrected
6. As far as ordering figures and table ordering, I have corrected it according to its proper orders

Generally, I highlighted all correction undertaken in accordance with the comment, suggestion and format of PLOS ONE.

Thank you
